# Supplementary material for: IL-7-Adjuvanted Vaginal Vaccine Elicits Strong Mucosal Immune Responses in Non-Human Primates
Source: Front Immunol. 2021 Feb 25;12:614115. doi: 10.3389/fimmu.2021.614115 (PMC7947860; doi:10.3389/fimmu.2021.614115)
Supplement: Supplementary file 1 [file DataSheet_1.pdf]

Supplementary Material

SUPPLEMENTARY MATERIALS AND METHODS

Immunohistofluorescent staining.

Four-µm thick tissue sections fixed in formaldehyde, and embedded in paraffin (FFPE), collected on glass slides (SuperFrost ® Plus, Menzel-Gläser, Illkirch, France) were dewaxed and rehydrated. Unmasking of the antigens was carried out by pressure cooking the sections for 15 minutes in 0.01M buffered sodium citrate solution (pH 6) or for 20 minutes in 1mM EDTA/10mM Tris Buffer (pH 9). Sections were then rinsed with PBS.

Eight-µm thick cryosections were collected on glass slides (SuperFrost® Plus), air dried overnight, fixed with 100% acetone for 10 minutes at room temperature (RT), air dried for 4 hours then stored at -80°C until use. Before immunostaining, frozen sections were fixed for 5 minutes at RT in 50/50 acetone/methanol and rinsed with PBS, excepted for chemokine staining.

Both types of sections were incubated for 30 minutes with blocking buffer (2% normal goat serum and 5% BSA in PBS or 5% BSA in PBS when using primary goat antibodies), incubated overnight with primary antibodies at 4°C, washed in PBS/0.5%Tween20 (Sigma-Aldrich) at RT, incubated with secondary antibodies in the dark for 30 minutes at RT and washed in PBS/0.5%Tween20. Finally, sections were washed in PBS alone, counterstained with 4,6-diamidino-2-phenylindol/DAPI (Molecular Probes, Cergy Pontoise, France) and mounted in Fluoromount-G (Southern Biotechnology, Birmingham, England). The antibodies used for immunohistofluorescence labeling are listed in Supplementary Table 2. For staining on FFPE sections, a supplemental 10 minutes incubation in 10mM $CuSO_4$/50mM NH_4Cl (pH 5) at RT was added after the DAPI step to reduce autofluorescence. For CD127 staining on FFPE sections, a 30 minutes incubation step in 2% normal goat serum, 5% BSA, 10% FcR Blocking Reagent (Miltenyi Biotec, Paris, France) in PBS was added followed by an amplification step with a biotin-conjugated secondary antibody and a streptavidin conjugated to Alexa Fluor® 488, 546 or 633 (Molecular Probes) after having used a Streptavidin/Biotin Kit according to manufacturer's recommendations (Vector Laboratories, Nanterre, France). OPAL™ kit was used according to manufacturer's recommendations (PerkinElmer®, Courtaboeuf, France), to detect anti-CD127 in combination with CD3 and MHC-II MamuLa-DR. Of note, anti-CD127 was incubated before anti-CD3 and MHC-II MamuLa-DR, which were then detected with Alexa Fluor®-conjugated secondary antibodies (Molecular Probes).

Quantification of total and DT-specific IgGs and IgAs by enzyme-linked immunosorbent assay.

Total and DT-specific immunoglobulins (IgGs or IgAs) were quantified in CVLs using an in house ELISA. For total Ig quantifications, Nunc™ MaxiSorp™ ELISA plates were coated with dilutions of samples and several dilutions of Human IgA (Jackson ImmunoResearch, West Grove, USA; 400 to 0.8 ng/mL) or Monkey IgG (Rockland Immunochemicals, Gilbertsville, USA; 400 to 0.8 ng/mL) standards and incubated overnight at 4°C, washed in PBS/0.1%Tween20, blocked with 2% BSA in PBS/0.1%Tween20 for 2 hours at 37°C, washed in PBS/0.1%Tween20, then incubated with horseradish peroxidase (HRP) conjugated goat anti-monkey IgA (Abnova, 0.1ug/mL) or IgG antibodies (Rockland Immunochemicals;

0.07ug/mL) for 1 hour at 37°C. After several washes in PBS/0.1%Tween20, 3,3',5,5'-Tetramethylbenzidine/TMB peroxidase substrate solution was added (KPL, Maryland, USA) and incubated for 10 minutes at RT, then the reaction was stopped with 1M of H_3PO_4. Plates were read at 450nm (SpectraMaxTM384 PLUS ELISA Microplate Reader, Molecular Devices) and duplicate experiments were performed for each sample. Data were analyzed using SoftMax Pro software (5.0.1 version, Molecular Devices). Total IgA or IgG concentrations were determined by interpolation, using the calibration line of IgA or IgG standards, respectively. For quantification of specific Igs, ELISA plates were coated with DT (1µg/mL) and incubated overnight at 4°C, washed in PBS/0.1%Tween20, blocked with 2% BSA in PBS/0.1%Tween20 for 2 hours at 37°C, incubated with dilutions of each samples overnight at 4°C, washed in PBS/0.1%Tween20, then goat anti-monkey IgA- (Abnova) or IgG-HRP conjugated antibodies (Rockland Immunochemicals) were added for 1 hour at 37°C. Finally, after several washes in PBS/0.1%Tween20, peroxidase substrate was added (TMB from KPL) and incubated for 30 minutes at 37°C and the reaction was stopped with 1M of H_3PO_4. Plates were read and analyzed as mentioned above. Samples with a signal at least twice above the background were considered positive. Total IgA or IgG concentrations were determined by interpolation, using serial dilutions of monkey IgGs (Rockland Immunochemicals, Gilbertsville, USA) or human IgAs (Jackson ImmunoResearch, West Grove, USA). In contrast, the absolute quantification of the concentration of DT-specific IgAs and IgGs cannot be performed due to the lack of commercially available purified monkey DT-specific IgGs and IgAs that could be used as standards. Accordingly, we normalized DT-specific IgG and IgA data at each dilution (1/d) on the concentrations of IgGs and IgAs (mg/mL) in the CVLs and expressed DT-specific IgGs or IgAs in the CVLs as OD×d/[Ig]mg/mL (3 dilutions per sample; in duplicate experiments). For each sample, data are presented for the first dilution (1/d) giving an OD at least twice above the background. In addition, a few samples were systematically tested in all the ELISA plates in order to normalize the results obtained in the different experiments. Results are expressed as OD for specific anti-DT divided by the concentration of total IgA or IgG (mg/mL) in a given sample.

Preparation of cells for ELISPOT assay.

Iliac LN and vagina were samples at necropsy from macaques of the IL-7+DT and PBS+DT groups. LN cells were isolated by crushing the organs through a 40 µm cell strainer to obtain a single cell suspension. The cells were then washed in RPMI 1640 medium containing 100 U/mL penicillin, 100 µg/mL streptomycin and 10% fetal calf serum (RPMI/PS/10% FCS), centrifuged, and cell numbers and viability were determined by trypan blue exclusion.

Vaginal cells were prepared from the upper region of the vaginal walls and from the fornix. Small pieces of tissues (<10mm^3) were incubated in RPMI/PS/Hepes 20mM supplemented with dispase II (2.6 U/ml, Sigma) 2h at 37°C, then washed in RPMI/PS/Hepes 20mM supplemented with 5mM EDTA, then in RPMI/PS/Hepes 20mM and each time the supernatant was removed through a 100µm cell strainer. The tissue pieces were then digested in RPMI/PS supplemented with collagenase VIII (2 mg/mL, Sigma) and DNase (20 U/mL, ROCHE) for 1h30 on a magnetic stirrer at 37°C, the supernatant decanted through a 100µm cell strainer was saved, and the collagenase/DNase treatment was repeated once with fresh medium. The cells were washed in RPMI/PS/10% FCS, passed through 70µm then 40µm cell strainers, centrifuged, and the numbers and viability of cells were determined by trypan blue exclusion.

Quantification of antibody secreting cells by B-cells ELISPOT.

Antibody secreting cells (ASCs) were assayed in Multiscreen HA plates (Merck Milipore, Molsheim, France) coated with DT (10µg/mL), overnight at 4°C. After washes with PBS, the

reactive sites were blocked by incubation with RPMI/PS/10% FCS for 2 hours at 37°C. After washes, PBMCs or cell suspensions recovered from tissue were transferred into plates at 1.10^6, 4.10^5, 1.10^5 cells per well in RPMI/PS/10% FCS and incubated for 40 hours at 37°C in 5% CO_2. After removal of the cells, the plates were washed again and the DT-specific antibodies were detected with either goat anti-monkey IgG-HRP (Rockland Immunochemicals) or goat anti-monkey IgA-HRP (Abnova) and incubated 4 hours at 37°C. Plates were washed and spots were detected by addition of AEC (ImmPACT AEC, VECTOR Laboratories). Spots were counted in an ELISPOT Reader (BIOREADER®-5000-F, BioSys GmbH, Serlabo, Entraigues, France). Spot numbers were reported as DT-specific ASCs per million of PBMCs.

SUPPLEMENTARY FIGURES

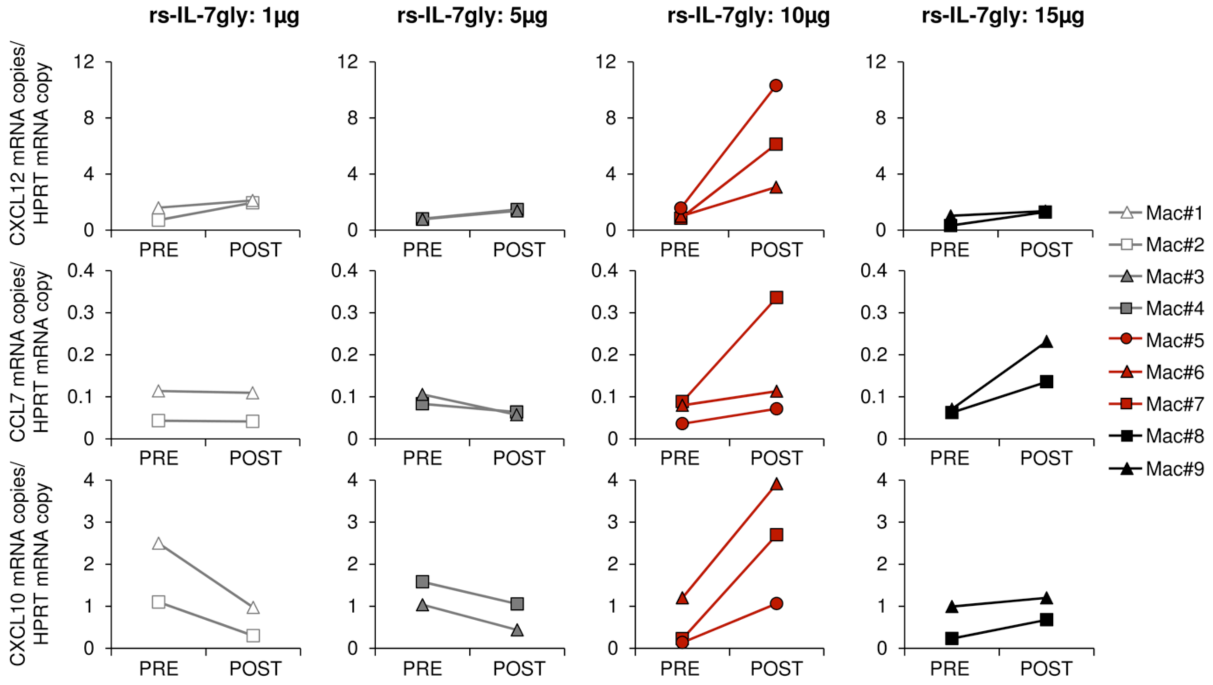

Supplementary Figure 1. Topical rs-IL-7gly administration induces local chemokine transcription in the vaginal mucosa.

The transcription of CXCL12 (top panels), CCL7 (middle panels) or CXCL10 (bottom panels) was quantified in vaginal biopsies sampled from macaques (n=9) one month before (PRE) and 48 hours after (POST) administration, by vaginal spray, of 1µg (light gray symbols), 5µg (dark gray symbols), 10µg (red symbols) or 15µg (black symbols) of rs-IL-7gly. Each point represents the median of 4 vaginal biopsies. For each sample, the data were normalized to HPRT mRNAs simultaneously quantified with the chemokines.

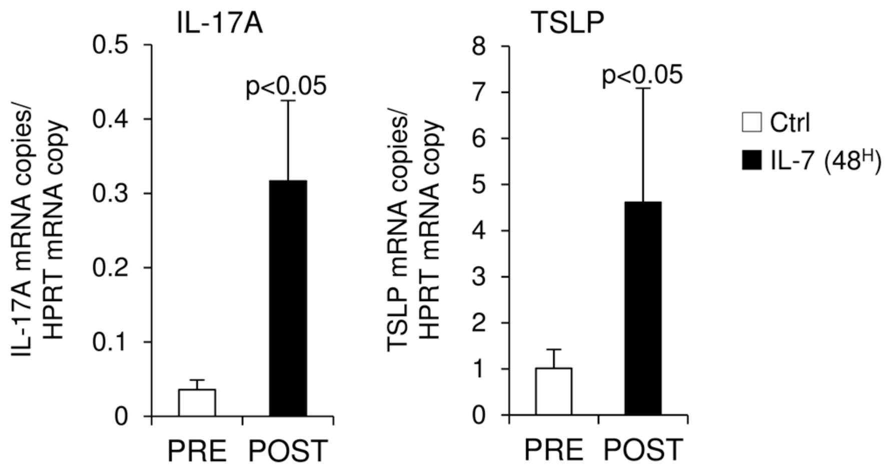

Supplementary Figure 2. Topical administration of rs-IL-7gly induces local transcription of cytokines in the vaginal mucosa.

The transcription of IL-17A and TSLP (Thymic stromal lymphopoietin) was quantified in vaginal biopsies (n=4 per macaque and time point) taken from macaques (n=5) one month before (Ctrl, white bars) and 48 hours after (IL-7 48^H, black bars) the administration of 10µg (n=3 macaques) or 15µg (n=2 macaques) of rs-IL-7gly, by vaginal spray, and normalized to HPRT mRNAs quantified simultaneously with the cytokines (mRNA copies/HPRT mRNA copy). Means and SEM of the data obtained for each of the 5 macaques are presented. Statistical differences are shown (Wilcoxon Signed-Rank Test).

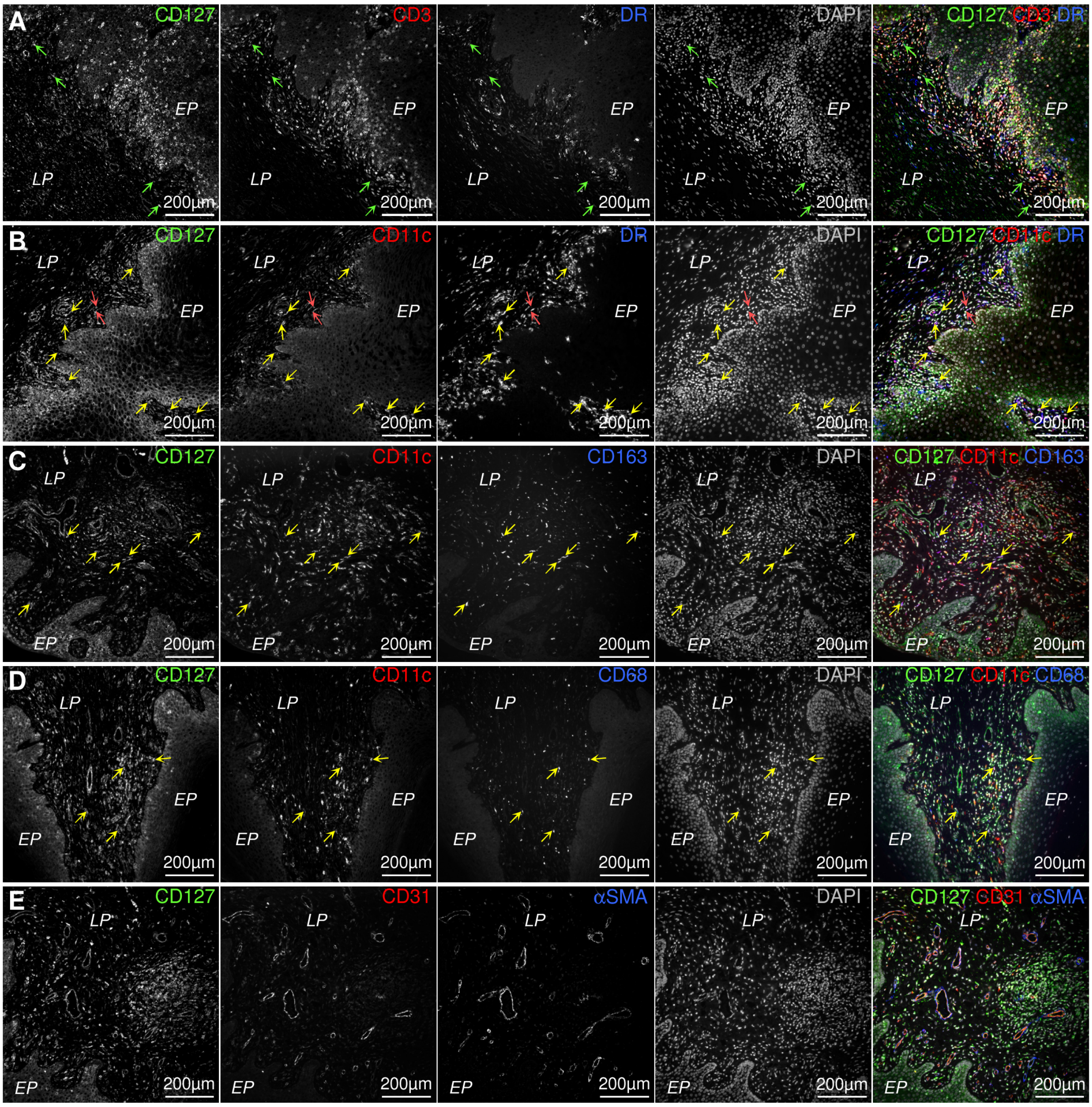

Supplementary Figure 3. CD127-expressing cells in the vaginal mucosa.

Sections of vaginal mucosa sampled from healthy monkeys were stained with anti-CD127 antibodies (**A-E**; green) in combination with anti-CD3 (**A**; red), anti-MamuLa-DR (**A, B**; blue), anti-CD11c (**B, C, D**; red), anti-CD163 (**C**; blue), anti-CD68 (**D**; blue), anti-CD31 (**E**; red) and/or anti-αSMA (**E**; blue) antibodies. Nuclei were stained with DAPI (grey). *Green arrows identify $CD127^{+}CD3^{-}MamuLa\text{-}DR^{-}$ cells; Yellow arrows identify triple positive cells: (B) $CD127^{+}CD11c^{+}MamuLa\text{-}DR^{+}$, or (C) $CD127^{+}CD11c^{+}CD163^{+}$, or (D) $CD127^{+}CD11c^{+}CD68^{+}$; Red arrows identify $CD127^{+}CD11c^{+}MamuLa\text{-}DR^{-}$ cells; EP: Pluristratified Epithelium; LP: Lamina Propria. DR: MHC-II MamuLa-DR.*

A – IL-7R and Vaginal Epithelial Cells

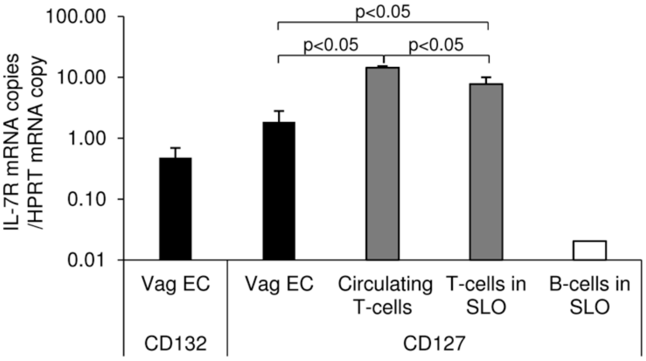

B – Adherent Vaginal Epithelial Cells in culture

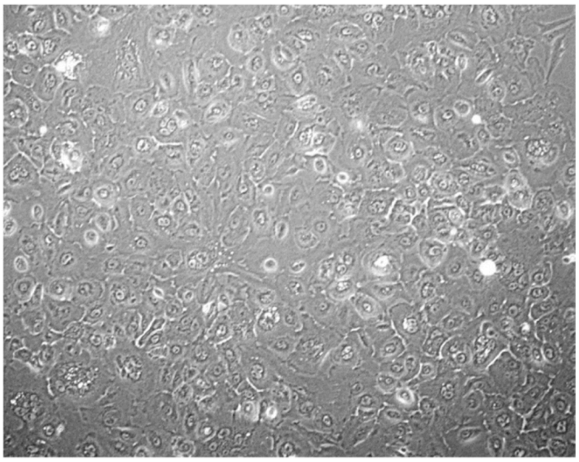

Supplementary Figure 4. IL-7R and Vaginal Epithelial Cells.

(A) mRNAs coding for CD132 (γc chain) and CD127 (IL-7Rα chain) were quantified in primary vaginal epithelial cells (Vag EC) obtained after Dispase/EDTA treatment of the vaginal epithelial layer, circulating T-cells, T-cells and B-cells purified from secondary lymphoid organ (SLO: spleen and lymph node) gathered from 3 healthy macaques and were normalized to HPRT mRNAs simultaneously quantified together with IL-7R mRNAs (IL-7R mRNA copies/HPRT mRNA copy). Bars and error bars represent means and SEM, respectively. Statistically significant differences are shown (Mann-Whitney U test). **(B)** Example of primary simian vaginal epithelial cells cultured for 2 weeks after isolation, on flask coated with collagen I in SmGM2 medium (Lonza).

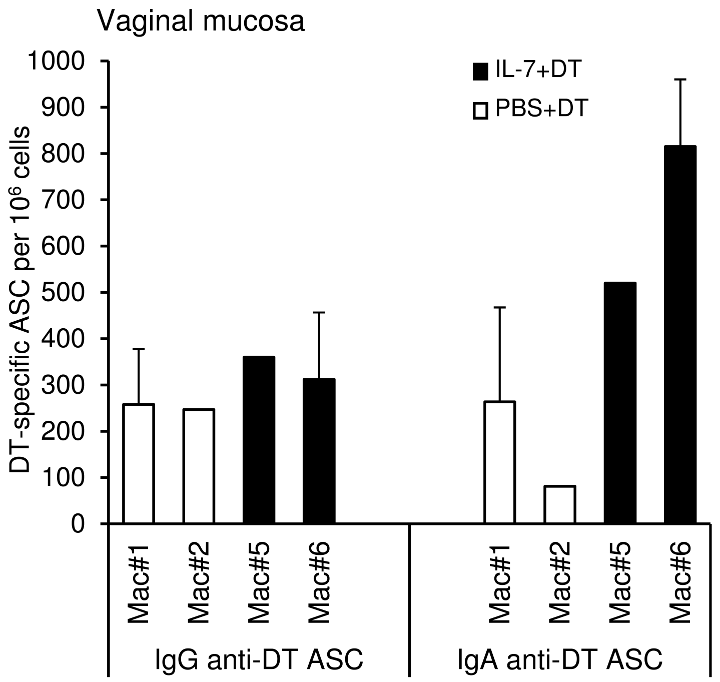

Supplementary Figure 5. Preferential localization of DT-specific IgAs plasma cells in the vaginal mucosa following rs-IL-7gly-adjuvanted mucosal immunization.

IgG- and IgA-producing DT-specific plasma cells (ASC) were quantified by B-cell ELISPOT on isolated cells from the vaginal upper part and the vaginal fornix of macaques immunized with PBS+DT (White bars, n=2 macaques) or IL-7+DT (Black bars, n=2 macaques), sampled at necropsy. Results are expressed as IgG or IgA anti-DT-specific plasma cells per 10^6 cells. Bars and error bars represent means and SD, respectively (4 replicates for Mac#1 and Mac#6, one replicate for Mac#2 and Mac#5). *ASC: antibody secreting cells.*

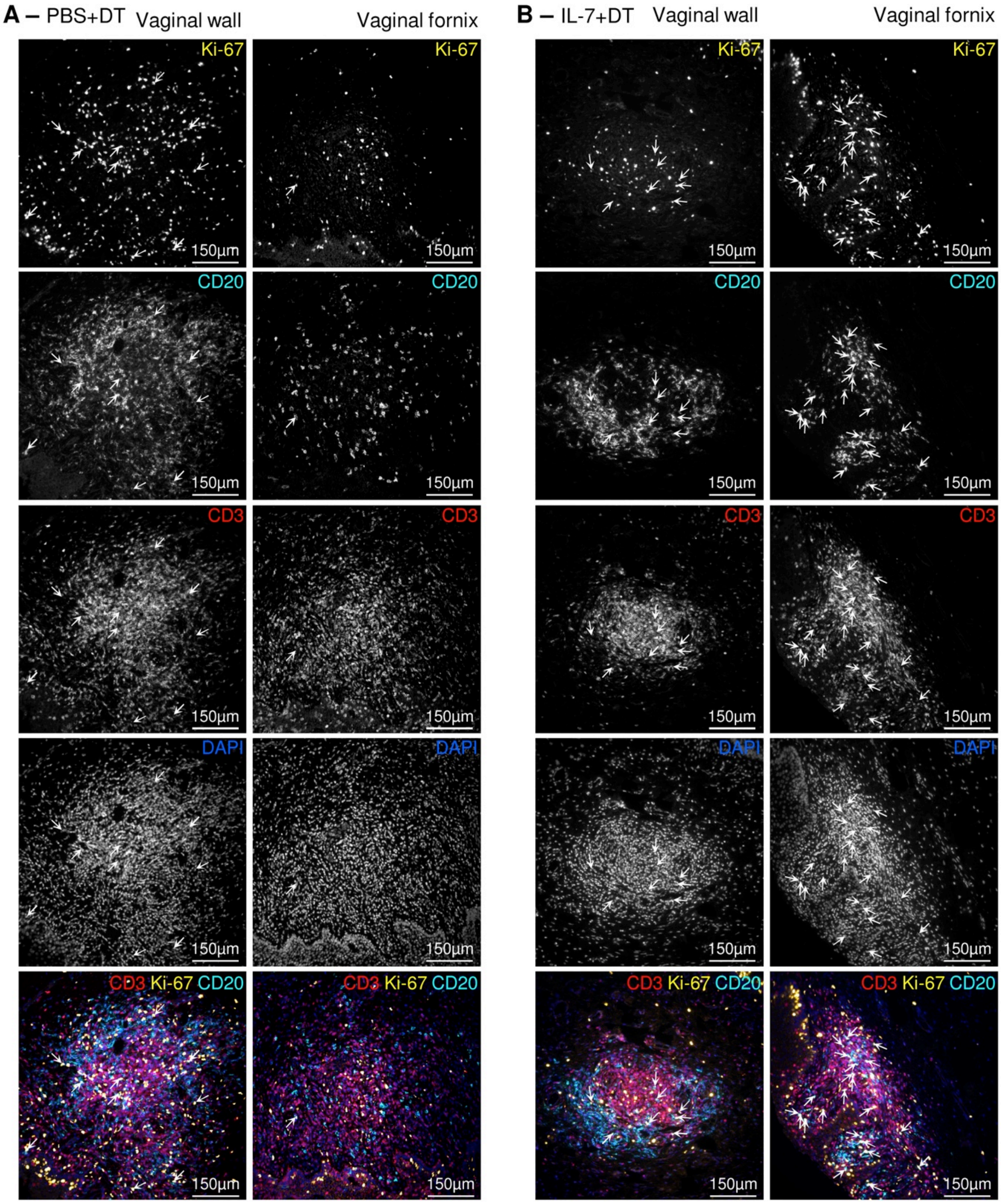

Supplementary Figure 6. More B-cells proliferate in ectopic lymphoid follicles in the vaginal mucosa of IL-7-treated DT-immunized macaques.

Sections of vaginal walls (left panels; vaginal upper part) and vaginal fornix (right panels) gathered from PBS+DT **(A)** and IL-7+DT **(B)** -immunized macaques at necropsy were labeled with anti-Ki-67 (yellow), anti-CD3 (red) and anti-CD20 (cyan) antibodies. Nuclei were stained with DAPI (blue). Arrows identify all B-cells expressing Ki-67.

SUPPLEMENTARY TABLES

Supplementary Table 1. Oligonucleotides used for real-time PCR quantification of chemokines, cytokines and IL-7R mRNA.

**Outer 3'/5' primer pairs for first amplification**		**Inner 3'/5' primer pairs for qPCR**	
**Name**	**Sequence**	**Name**	**Sequence**
HPRT-Out5	CTGAACGTCTTGCTCGAGAT	HPRT-In5	CACATTGTAGCCCTCTGTGT
HPRT-Out3	CGACCTTGACCATCTTTGGA	HPRT-In3	CTGACCAAGGAAAGCAAAGT
CCL2-Out5	AACATCCAGTGCTCAAACTGAA	CCL2-In5	CTGCTCATAGCAGCCACCTTCA
CCL2-Out3	TCCAGGTGGTCCATGGAAT	CCL2-In3	TCCTGAACCCACTTCTGCTT
CCL3-Out5	CATTCCATCACCTGCTCAGAA	CCL3-In5	CAACCGGATCTCAGCAACAT
CCL3-Out3	TCCAGGTCGCTGACGTATTT	CCL3-Int3	GCCGGCCTCTCTTGGTTA
CCL4-Out5	CCATGAAGCTGCGCGTGA	CCL4-In5	CCCACCTCCTGCTGCTT
CCL4-Out3	CTCAGTTCAGTTCCAGGTCA	CCL4-In3	GCAGACTTGCTTCCCTCTTTT
CCL5-Out5	GCTGTCATCCTCGTTGCTA	CCL5-In5	ACATGCCTCAGACACCACA
CCL5-Out3	CTCATCTCCAAAGAGTTGATGTA	CCL5-In3	TACTCCCGAACCCATTTCTT
CCL7-Out5	CATGCCCTCACCCTCCA	CCL7-In5	CATGCCCTCACCCTCCA
CCL7-Out3	CTCTGAGAAAGGACAGGGTA	CCL7-In3	ATTTGTTTCTCAAGTCATGGCTT
CCL8-Out5	GGACTTGCTCAGCCAGATT	CCL8-In5	TGCTCAGCCAGATTCAGTTT
CCL8-Out3	GGTCCAGATGCTTCATGGA	CCL8-In3	CCTGACCCATCTCTCCTT
CCL11-Out5	TGAAGGTCTCCACAACACT	CCL11-In5	TGCAACCACCTGCTGCTTTA
CCL11-Out3	TGGCTTTGGAGTTGGAGAT	CCL11-In3	TTGGAGATTTTCGGTCCAGATA
CCL17-Out5	GCAGCTCGAGGGACCA	CCL17-In5	GCAGCTCGAGGGACCA
CCL17-Out3	TCAAGGCTTTGCAGGTATTTAA	CCL17-In3	GTTGGGGTCCGAACAGAT
CCL19-Out5	ACCGTTGGCCTGCCTCTGTT	CCL19-In5	CAGCCTGCTGGTTCTCTGGA
CCL19-Out3	CTGCTGCGGCGCTTCATCTT	CCL19-In3	TCCTCTGCAGTCTCTGGAT
CCL20-Out5	CCATGTGCTGTACCAAGAGT	CCL20-In5	TTGCTCCTGGCTGCTTTGA
CCL20-Out3	TGCTGAGGCGACGTACAATA	CCL20-In3	CCCAGGTCTGCTTTGGATT
CCL21-Out5	CCTCAGCTCTGGCCTCTTA	CCL21-In5	ATGGCTCAGTCACTGGCTCT
CCL21-Out3	TCACTGGGCTATGGCCCTTTA	CCL21-In3	CTATGGCCCTTTAGGGGTCT
CCL22-Out5	CATGGCTCGCCTACAGA	CCL22-In5	GTGACGCTTCAAGCAACTGA
CCL22-Out3	GGGATGGAGATCAGGGAAT	CCL22-In3	GATCAGGGAATGCAGAGAGT
CCL25-Out5	CACACCCAAGGTGTCTTTGA	CCL25-In5	TTATCGGATCCAGGAGGTGA
CCL25-Out3	TTAGCTGATGTCAGGAGGGA	CCL25-In3	CTGCTGGTGGGATTGCTAAA
CCL28-Out5	AAGGCTCCTGGAAAGAGTGAA	CCL28-In5	TCGCATCCAGAGAGCTGAT
CCL28-Out3	GTTTCGTGTTTCCCCTGATGT	CCL28-In3	CTGATGTGCCCTGTTACTGT
CXCL8-Out5	GAACCATCTCGCTCTGTGTA	CXCL8-In5	ATCTCGCTCTGTGTAAACATGA
CXCL8-Out3	GCTCTCTTCCATCGGAAAGT	CXCL8-In3	CTGTATTGGCACAGTGTGGT
CXCL10-Out5	TGATTTGCTGCCTTGTCTTTCT	CXCL10-In5	CTCTCTCAAGAACTGTACGCT
CXCL10-Out3	ACCTCTTCTCACCCTTCTTTT	CXCL10-In3	CATGTGGACAAAATTGACTTGGA
CXCL12-Out5	GTCAGCCTGAGCTACAGAT	CXCL12-In5	TACAGATGCCCATGCCGATT
CXCL12-Out3	TTTCTCCAGGTACTCCTGAA	CXCL12-In3	TCGGGTCAATGCACACTTGT
CXCL13-Out5	TGCTTCTCATGCTGCTGGT	CXCL13-In5	TCTCTCCAGTCCAAGGTGT
CXCL13-Out3	AACACTGGAACTGGTGGAGT	CXCL13-In3	GGAACTGGTGGAGTTGAAGA
CX3CL1-Out5	TATCTCTGTCGTGGCTGCT	CX3CL1-In5	GTCCTGCTGGCTGGACA
CX3CL1-Out3	CCTCCATCCTGAGCCTTT	CX3CL1-In3	TCCCTGAGGAACCAGTCA
IL-17A-Out5	GCCATAGTGAAGGCAGGAAT	IL-17A-In5	GCCATAGTGAAGGCAGGAAT
IL-17A-Out3	AAGCCTAACTGCTTTGGGGA	IL-17A-In3	AGTATCTTCTCCAGCCGGAA

IL-21-Out5	CGTCTAGCTCTACTGTTGGT	IL-21-In5	AGTCCTGGCAACATGGAGA
IL-21-Out3	CAAGTTAGATCCTCAGGAATCTT	IL-21-In3	CTAGAGGACAGATGCTGATGA
TSLP-Out5	CTTTCAACTTGTAGGGCTGGT	TSLP-In5	CTTTCAACTTGTAGGGCTGGT
TSLP-Out3	TGTGACACTTGTTCCAGACATTT	TSLP-In3	CTCTTCTTCATTGCCTGAGTA
LTb-Out5	GATCAGGGAGGACTGGTAA	LTb-In5	CAACAAGGACTGGGGTTTCA
LTb-Out3	CGACGAGACAGTAGAGGTAA	LTb-In3	CGACGAGACAGTAGAGGTAA
LTa-Out5	CGTCAGCACCCCAAGAT	LTa-In5	CAGAACTCACTGCTCTGGA
LTa-Out3	ACAGTACTAGGGCTGAGGA	LTa-In3	CCATCTGTGTGGGTGGATA
CD127-Out5	TGCGCAAAATGGAGACTTGGA	CD127-In5	GCCAGTTGGAAGTGAATGGA
CD127-Out3	GTCATTGGCTCCTTCACGAT	CD127-In3	TCAAAAGGAGCCTCAGGTTTAA
CD132-Out5	CAGAAGTGCAGCCACTATCTA	CD132-In5	CAGAAGTGCAGCCACTATCTA
CD132-Out3	TTGTTCAGTCCAGCTGTGGT	CD132-In3	GTGCTCCAAACAGTGGTTCA

Supplementary Table 2. Antibodies used for the immunostaining.

**Primary antibodies**				
**Target epitope, cell type, chemokine**	**Isotype**	**Clone**	**Supplier**	**Supplier details**
CD3 [a,b,c]	Rabbit IgG	(polyclonal)	DAKO	Trappes, France
CD4 [a]	Mouse IgG1	L200	BD Biosciences	Le Pont de Claix, France
CD8 [a]	Mouse IgG1	RPA-T8	BD Biosciences	Le Pont de Claix, France
CD20 [a,b,c]	Mouse IgG2a	L26	DAKO	Trappes, France
CD31 [a,b,c$]	Mouse IgG1	JC70A	DAKO	Trappes, France
PNAd [b,c]	Rat IgM	MECA-79	Biolegend	St-Quentin en Yvelines, France
GL-7 [b,c]	Rat IgM	GL-7	Biolegend	St-Quentin en Yvelines, France
HLA-DR [a,b]	Mouse IgG1	TAL.1B5	DAKO	Trappes, France
Tissue macrophages [a]	Mouse IgG1	PM-2K	AbD Serotec	Düsseldorf, Germany
DC-SIGN [a]	Mouse IgG2b	120507	R&D systems	Lille, France
CD11c [a]	Mouse IgG1	3.9	BioLegend	St-Quentin en Yvelines, France
CD11c [b]	Mouse IgG2a	5D11	LeicaBiosystems	Nanterre, France
CD68 [b]	Mouse IgG1	KP1	DAKO	Trappes, France
CD163 [b,]	Mouse IgG1	10D6	Novacastra	Nanterre, France
αSMA [b,c$]	Mouse IgG2a	1A4	ThermoFischer	Villebon sur Yvette, France
CD83 [a]	Mouse IgG2b	HB15A	Beckman Coulter	Marseille, France
CD127 (C-ter) [b]	Rabbit	(polyclonal)	Abcam	Paris, France
Ki-67 [b]	Mouse IgG1	B56	BD Biosciences	Le Pont de Claix, France
CCL2 [a]	Rabbit	(polyclonal)	Aviva Systems Biology	San Diego, USA
CCL5 [a]	Rabbit	(polyclonal)	Aviva Systems Biology	San Diego, USA
CCL7 [a]	Rabbit	(polyclonal)	Aviva Systems Biology	San Diego, USA
CCL19 [a]	Mouse IgG2b	54909	R&D systems	Lille, France
CXCL13 [a]	Goat	(polyclonal)	R&D systems	Lille, France
CXCL12 [a]	Mouse IgG1	79018	R&D systems	Lille, France
IgG [a]	Rabbit	(polyclonal)	DAKO	Trappes, France
IgA [a]	Rabbit	(polyclonal)	DAKO	Trappes, France
Anti-DT-FITC [a]	Goat IgG	(polyclonal)	Abcam	Paris, France
**Secondary antibodies**				
**Target species**	**Host species**	**Conjugate**	**Supplier**	**Supplier details**
Mouse (IgG1, 2a or 2b)	Goat	Alexa Fluor® 546, 488 or 633	Molecular Probes	Cergy Pontoise, France
Rabbit	Goat	Alexa Fluor® 546, 488 or 633	Molecular Probes	Cergy Pontoise, France
Goat	Chicken	Alexa Fluor® 488	Molecular Probes	Cergy Pontoise, France
Rabbit	Donkey	Alexa Fluor® 546	Molecular Probes	Cergy Pontoise, France
Rabbit	Goat	Biotin	DAKO	Trappes, France
Goat	Donkey	Biotin	Abcam	Paris, France

The tissue sections used for immunohistofluorescence labeling were [a] cryopreserved, [b] formaldehyde-fixed paraffin-embedded (FFPE) with acidic antigen retrieval (0.01M sodium citrate buffer, pH 6.0) or [d] FFPE with basic antigen retrieval (1mM EDTA/10mM Tris Buffer, pH 9.0). [$] Cell staining was much better and reproducible with basic antigen retrieval as compare as acidic antigen retrieval.
